# Severe head injury in children: Management of a traumatic craniotomy case in a resource-constrained hospital

**DOI:** 10.1016/j.ijscr.2024.109883

**Published:** 2024-06-14

**Authors:** Tamassi Bertrand Essobiyou, Albert Kossi Labou, Samuel Salem Laurent Ouedraogo, Pegwende Rachid Cedric Diendere, Stéphane Essossinam Kpelao

**Affiliations:** a General Surgery Department, Sylvanus Olympio University Hospital Center, Lome, Togo; b General Surgery Department, Dapong Regional Hospital Center, Dapaong, Togo; c Neurosurgery Department, Sylvanus Olympio University Hospital Center, Lome, Togo; d Anaesthesia and Reanimation Department, Charles de Gaulle Hospital, Ouagadougou, Burkina Faso; e Radiology Department, Charles de Gaulle Hospital, Ouagadougou, Burkina Faso

**Keywords:** Wound, Trauma, Craniocerebral, Craniotomy, Child

## Abstract

**Introduction and importance:**

Craniocerebral wounds are potentially serious and life-threatening injuries. These are real medical and surgical emergencies. The authors report a case of craniocerebral injury in a child with extensive craniotomy and its management in a hospital with limited resources in Togo.

**Case report:**

He was a young 11-year-old schoolboy who presented with an extensive craniocerebral injury with craniotomy after a road traffic accident. On admission, he had no focal neurological deficits or other signs related to an intracranial expansive process. After preoperative reanimation, antibiotic therapy and anti-tetanus serovaccination, he was taken to the operating room by general surgeons. He underwent lavage, suture of the dura mater, placement of the bone flap and suture of the scalp wound. The postoperative course was simple.

**Clinical discussion:**

Cranioencephalic trauma is one of the main causes of pediatric mortality in developing countries. Cranio-cerebral wounds are a therapeutic emergency because of the risk of infection, which remains the main concern. Treatment consists of a medical component followed by a surgical component. Reanimation remains an essential component of medical treatment.

**Conclusion:**

Craniocerebral wounds are serious injuries. It requires rapid and appropriate medical and surgical management to avoid complications, particularly infection.

## Highlights

• Craniocerebral wounds are serious injuries.
• These injuries are associated with a high mortality rate and a high risk of after-effects.
• Treatment requires appropriate technical facilities and is difficult in developing countries.
• Good management of the initial phase can improve vital prognosis.
• This initial management is now facilitated by the Advanced Trauma Life Support (ATLS) procedure.

## 1 Introduction

Craniocerebral wounds are rare, serious and life-threatening head injuries [1,2]. A craniocerebral wound is any injury characterized by an effraction of the skin, reaching the dura mater with contact between the brain and the external environment [[1], [2], [3]]. They are medical and surgical emergencies, both diagnostic and therapeutic [[1], [2], [3]]. The seriousness of these wounds stems from the risks to which they are exposed, particularly the risks of haemorrhage, infection and neurological sequelae [1,2]. Formerly the preserve of armed conflicts, cranioencephalic wounds are of increasingly diverse origin with the expansion of the road fleet and the road accidents that accompany it [1,2]. While depressed skull fractures are quite often encountered during these injuries, a true craniotomy is rare to exceptional [3]. We did not find any case reported in the literature. We report a case of craniocerebral injury with craniotomy in a young schoolboy during a road accident and its management in a regional hospital in Togo. The work has been reported in line with the SCARE 2023 criteria [4].

## 2 Case report

**Fig. 1:**
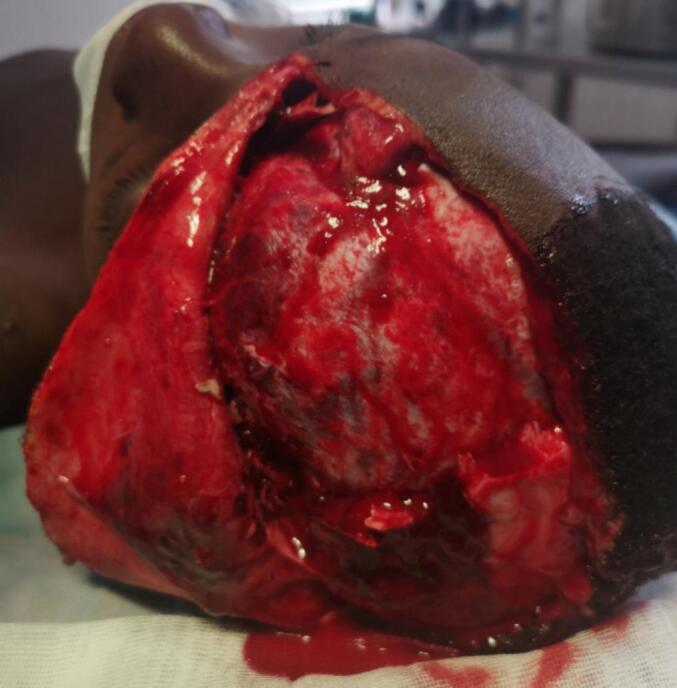
Clinical aspect of the craniocerebral wound.

**Fig. 2:**
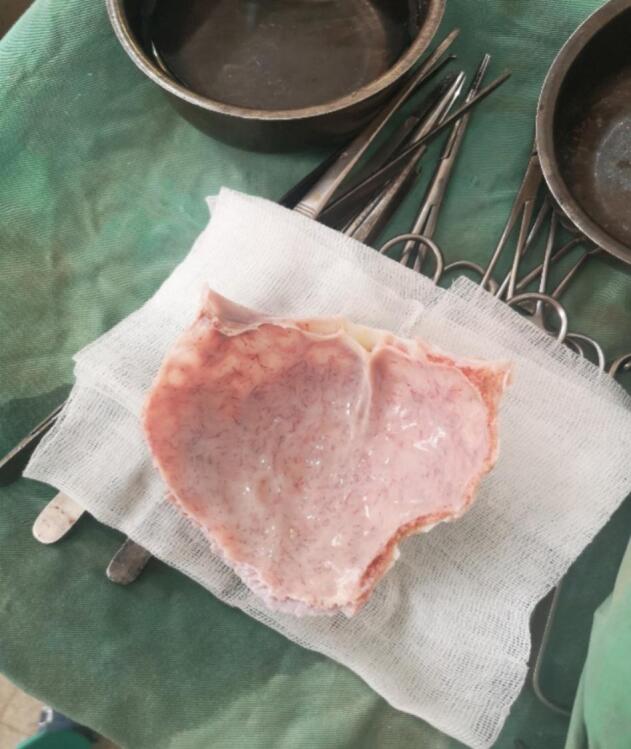
Operative view of the cranial flap before its installation.

He was an 11-year-old schoolboy, with no reported history, who was admitted in an emergency with a lucid interval from an open head injury. The duration of the loss of consciousness was not assessed. The trauma occurred during a public road activity with a pedestrian-automobile impact. The patient had not received previous tetanus prevention. On admission, an initial examination was performed using the ATLS (Advanced Trauma Life Support) procedure. The patient's airway was clear and he was breathing heavily. The patient was conscious and the examination of the cervical spine revealed no sign of. There was patchy bleeding from the scalp, prompting the application of an occlusive dressing for haemostatic purposes. Blood pressure and pulse were normal. No neurological deficit was noted. Completion of the examination ruled out a haemodynamic shock (Class I of ATLS). Following examination of the patient, a left frontoparietal wound with scalp detachment was noted. In addition, there was a frontoparietal craniotomy with cerebral exposure (Fig. 1). The intact bone fragment was delivered by the parents. It was largely intact and we packed it for the block in isotonic saline after washing. There was no objectified loss of brain substance; the brain surface was even. The entire wound was soiled with soil and road debris. The patient's examination revealed no focal neurological deficits or other signs of a possible intracranial expansive process. No imaging was performed. CT scans were not available in the area. Due to the soiling of the wound by soil and road debris, the wound was considered to have a high risk of infection. The patient received prophylactic antibiotic coverage with ceftriaxone and metronidazole. He also received tetanus and pneumococcal vaccination. Analgesic treatment included paracetamol and tramadol. We concluded that the patient had a left frontoparietal craniocerebral wound with a several scalp detachment but without loss of brain substance. However, we did not rule out an associated brain injury and indicated a parage-suture in the operating room. The preoperative workup was normal. Under general anaesthesia, we proceeded to a thorough washing of the wound with physiological serum about 6 h after admission. We noted a smeared bleeding wound, there was no depression. The surface was even. We noted a breach of the dura mater in the frontal area (about 1 cm) which we repaired with a simple suture after trimming the skin wound and a second wash. We proceeded with the placement of the detached cranial vault and finished by suturing the wound (Fig. 2). Management was performed by general surgeons; no neurosurgeons were available in the area. Post-operative management was straightforward with painkillers and antibiotics. The patient was discharged after three weeks. The follow-up at 6 months was satisfactory; no complications were detected.

## 3 Discussion

Cranioencephalic trauma is one of the main causes of pediatric mortality in developing countries [5]. The real incidence of these injuries is not known in Togo where they remain under-documented [5]. However, they are responsible for a significant socio-economic impact [5]. The main circumstances of occurrence of these injuries in children in developing countries are public road accidents and falls [5,6]. The literature reports a predominance of males, with boys being more prone to the causative factors [5]. Similarly, road traffic accidents are the preserve of older children, while falls predominate in other pediatric categories [5]. One explanation for this is the lack of responsiveness of younger children to protect their heads in simple falls [5].

Craniocerebral wounds are traumatic injuries characterized by triple damage: skin, bone and dura mater [1,2,7]. Their severity is related to haemorrhage, infection and the risk of neurological sequelae [1,2,6,7]. No previous study on these lesions in Togo was found in the literature. However, a study by Doleagbenou et al. in Lomé (Togo) reported that craniocerebral wounds were the second most common injury encountered during head trauma in children [5]. They are real medical and surgical emergencies [1,5,7].

Diagnostically, the clinic should systematically look for prognostic factors [1,2,7]. These factors are of great value in the therapeutic approach [1,2]. Respiratory and circulatory distress should be sought and corrected before neurological distress [1,2,7]. In addition, there is a strong relationship between the Glasgow score on admission and mortality [1,2,7]. After initial stabilization, an accurate assessment of the lesion involves the use of imaging techniques. Computed tomography is the first-line examination [[1], [2], [3],^7^]. It allows for more detailed exploration [1,2]. However, it is still difficult to access in certain areas, as was the case for us [1]. Standard radiography performed from the front and side could be an alternative [2,3,7]. It can confirm or deny a bone breach and motivate further exploration. In our case, the craniocerebral lesion was very expressive. However, we were not able to formally confirm the association of a parenchymal lesion, in particular a cerebral contusion, which is the most commonly reported associated lesion [1,2,7]. The predominance of frontoparietal location in craniocerebral wounds is unanimous in the literature [2,3,7].

Craniocerebral wounds are a therapeutic emergency [1,6,7]. The risk of infection is the main concern [[7], [8], [9]]. Any traumatic wound received in an emergency department should be considered infected [9]. Moreover, the presence of debris or foreign bodies is a considerable risk factor for infection [9]. The advent of broad-spectrum antibiotics has significantly reduced infectious events in craniocerebral wounds [1,2,7]. Management consists of a medical component followed by a surgical component. Medical treatment aims to stabilize hemodynamics and prevent infection [1]. It combines reanimation, broad-spectrum antibiotic prophylaxis, and anti-tetanus serovaccination [1,2,[7], [8], [9]]. Surgical treatment aims to blunt the communication between the brain and the external environment. Early surgical treatment considerably reduces the risk of infection [[1], [2], [3]]. Ideally, it should include a cutaneous, bony, meningeal and cerebral phase [1,7]. Dural plasty remains the key to success [1,3,7].

One misconception is that the prognosis for craniocerebral trauma in children is good because of their neuronal plasticity. The prognosis for recovery in children after such trauma is guarded, particularly if the lesions are diffuse, the child is young or if strategic areas are affected [10]. This is easily explained by the fact that young children have acquired skills, particularly in the minimus, at the time of the trauma [10,11].

The main issue in the prognosis of craniocerebral trauma in children is cognitive and behavioural impairment [10,12,13]. They are most often underestimated because of their insidious nature [10]. This underestimation may be even greater in Togo, where we have no specialists in pediatric neuropathology. In all cases, a particularly long follow-up period is necessary to accurately determine the real neurological sequelae [10,12]. Motor impairment, if present, has a better prognosis [10].

## 4 Conclusion

Craniocerebral wounds are rare but immediately serious head injuries. They constitute medical and surgical emergencies with a vital prognosis. These injuries occur in the context of traffic accidents and falls among children in developing countries. The diagnostic process remains difficult in some regions due to the lack of access to appropriate imaging techniques. Craniocerebral wounds present a high infectious risk. Prompt treatment coupled with the correct use of antibiotics is the key to a satisfactory outcome.

## Consent for publication

Written informed consent was obtained from the parent of the patient for publication of this case report and accompanying images. A copy of the written consent is available for review by the Editor-in-Chief of this journal on request.

## Ethical approval

It's not necessary.

## Funding

The authors did not receive any funding.

## Author contribution

All other authors contributed to the data collection. All authors read and approved the final manuscript.

## Guarantor

Tamassi Bertrand ESSOBIYOU, M.D., General surgery department, Sylvanus Olympio University Hospital Center, TOGO.

## Declaration of competing interest

The authors declare that they have no competing interests.

## Data Availability

Data sharing does not apply to this article, as no datasets were generated or analyzed during the current study.
